# Mitochondrial Dysfunction and Decreased Cytochrome *c* in Cell and Animal Models of Machado–Joseph Disease

**DOI:** 10.3390/cells12192397

**Published:** 2023-10-03

**Authors:** Filipa Almeida, Ildete L. Ferreira, Luana Naia, Daniela Marinho, Ana Catarina Vilaça-Ferreira, Marta D. Costa, Sara Duarte-Silva, Patrícia Maciel, A. Cristina Rego

**Affiliations:** 1 CNC-UC-Center for Neuroscience and Cell Biology, University of Coimbra, 3004-504 Coimbra, Portugal; filipa.almeida1194@gmail.com (F.A.); ildete.ferreira@cnc.uc.pt (I.L.F.); luana.naia@ki.se (L.N.); marinhodaniela@live.com.pt (D.M.); 2 IIIUC-Institute for Interdisciplinary Research, University of Coimbra, 3030-789 Coimbra, Portugal; 3 Life and Health Sciences Research Institute (ICVS), School of Medicine, University of Minho, 4710-057 Braga, Portugal; pg40729@alunos.uminho.pt (A.C.V.-F.); martacosta@med.uminho.pt (M.D.C.); sarasilva@med.uminho.pt (S.D.-S.); 4 ICVS/3B’s-PT Government Associate Laboratory, 4805-017 Guimarães, Portugal; 5 FMUC-Faculty of Medicine, University of Coimbra, 3000-548 Coimbra, Portugal

**Keywords:** Machado–Joseph disease, oxygen consumption, calcium handling, mitochondrial membrane potential, mitochondrial transcription

## Abstract

Mitochondrial dysfunction has been described in many neurodegenerative disorders; however, there is less information regarding mitochondrial deficits in Machado–Joseph disease (MJD), a polyglutamine (polyQ) disorder caused by CAG repeat expansion in the *ATXN3* gene. In the present study, we characterized the changes in mitochondrial function and biogenesis markers in two MJD models, CMVMJD135 (MJD135) transgenic mice at a fully established phenotype stage and tetracycline-regulated PC6-3 Q108 cell line expressing mutant ataxin-3 (mATXN3). We detected mATXN3 in the mitochondrial fractions of PC6-3 Q108 cells, suggesting the interaction of expanded ATXN3 with the organelle. Interestingly, in both the cerebella of the MJD135 mouse model and in PC6-3 Q108 cells, we found decreased mitochondrial respiration, ATP production and mitochondrial membrane potential, strongly suggesting mitochondrial dysfunction in MJD. Also, in PC6-3 Q108 cells, an additional enhanced glycolytic flux was observed. Supporting the functional deficits observed in MJD mitochondria, MJD135 mouse cerebellum and PC6-3 Q108 cells showed reduced cytochrome *c* mRNA and protein levels. Overall, our findings show compromised mitochondrial function associated with decreased cytochrome *c* levels in both cell and animal models of MJD.

## 1. Introduction

Machado–Joseph disease (MJD), also known as spinocerebellar ataxia type 3 (SCA3), is a late onset autosomal, dominantly inherited neurodegenerative disease characterized by a large atrophy of the cerebellum, but also affecting other brain regions, such as the brainstem, basal ganglia and spinal cord. MJD is considered the most common hereditary ataxia, causing mostly motor symptoms in patients. The first symptoms of this disease appear in adult age, at around 40 years of age, and slowly progress over time. The symptoms of this disease are highly heterogeneous, the most frequent being cerebellar ataxia, progressive external ophthalmoplegia, dysarthria, dysphagia, pyramidal signs, dystonia, rigidity and distal muscular atrophy [1]. This neurodegenerative disorder affects around 1–2 individuals per 100,000 people [2], being highly prevalent in the Portuguese Azores islands, particularly in Flores Island, where the disease affects 1 in 239 individuals [3]. MJD is caused by the expansion of an unstable CAG tract in exon 10 of the *ATXN3* gene encoding for ataxin-3 (ATXN3) and is responsible for C-terminal expanded polyglutamine (polyQ) stretch in mutant ATXN3 (mATXN3). Several cellular and molecular mechanisms have been implicated in MJD pathogenesis, namely transcriptional deregulation, impaired axonal transport, defective macroautophagy and ubiquitin–proteasome system, calcium dyshomeostasis and cell death ([4] for review). Despite active research, there are still no effective neuroprotective therapies for MJD and only symptomatic treatments are available [5].

Mitochondrial dysfunction has been described as a cellular mechanism in several neurodegenerative disorders, namely in Huntington’s disease (HD), the most prevalent polyQ disorder (e.g., [6]), but little is known about its impact on MJD pathogenesis. We have previously shown enhanced cellular susceptibility to irreversible complex (Cx) II inhibition with 3-nitropropionic acid, and moderate compromised mitochondrial Cx II in MJD mouse cerebellar cells and in fibroblasts derived from MJD patients [7]. The expression of ATXN3 fragment derived from calpain cleavage was also shown to affect mitochondrial function and degradation [8]. Moreover, expression of mATXN3 in cells expressing WT α-synuclein largely enhanced cellular oxidation status [9]. Additionally, reduced levels of glutathione and activity of mitochondrial enzymes were reported in cellular models of MJD, which were accompanied by decreased mitochondrial DNA (mtDNA) copy number in MJD cells and human samples [10]. Supporting these observations, we also observed decreased mtDNA copy number and accumulation of the 3876 bp mtDNA deletions throughout disease progression [11,12]. Interestingly, mass spectrometry data identified several ATXN3 (both normal and polyQ-expanded) interactors, with most of them being involved in protein quality control and mitochondria [13]. The interaction of three mitochondrial proteins (succinate dehydrogenase [ubiquinone] iron–sulfur subunit, cytochrome *c* oxidase assembly factor 7 and cytochrome *c* oxidase subunit NDUFA4) was stronger for mATXN3 when compared to wild-type protein. These novel findings were accompanied by altered mitochondrial morphology in polyQ-expressing cells [13]. Additionally, mitochondrial deficits were previously observed in a cellular model of MJD (neuroblastoma cells expressing ATXN3-78Q), showing increased sensitivity to rotenone, which suggested mitochondrial respiration defects. Moreover, in this study, far-infrared radiation exposure resulted in an improved mitochondrial phenotype in these cells through prevention of polyQ protein accumulation [14]. Furthermore, we showed that chronic treatment with creatine, a cellular energy buffer, modified disease progression and significantly improved motor function in a CMVMJD135 mouse model of MJD. Additionally, creatine was able to improve some neuropathological findings in the brain of these mice as well as restore the levels of VDAC1/Porin [15]. Taking advantage of a knock-in mouse model of MJD (Ki91), it has been proposed that metabolic and mitochondrial proteins are altered in the brain of these mice, a dysfunction attributed to the failure of energy metabolism detected in neonatal MJD cerebellar neurons [16]. Recently, mitochondrial dysfunction was also found in SCA3 patient fibroblasts, evidencing circular mitochondria, reduced oxidative phosphorylation (OXPHOS) complexes, and decreased ATP production and cell viability associated with dysregulation of parkin-VDAC1-mediated mitophagy [17]. Despite these and other findings, the cytotoxic mechanisms behind MJD pathogenesis remain elusive and the role of mitochondrial dysfunction in MJD pathogenesis has not been fully studied yet.

Thus, in the present study, we characterized the alterations in mitochondrial function and markers of biogenesis in two different MJD models, namely mitochondria isolated from the cerebellum and brainstem (two brain regions most affected in MJD) of CMVMJD135 transgenic mouse brains and a tetracycline-regulated PC6-3 cell line expressing *ATXN3* Q108, versus Q28 control cells. Our results show reduced function of mitochondrial respiratory chain that is apparently linked to decreased cytochrome *c* levels in these MJD models, constituting a valuable therapeutic target in future pre- and clinical- trials.

## 2. Materials and Methods

### 2.1. Materials

Roswell Park Memorial Institute’s (RPMI) medium containing hygromycin, doxycycline hyclate, carbonyl cyanide 4-(trifluoromethoxy) phenylhydrazone (FCCP), adenosine triphosphate (ATP), adenosine diphosphate (ADP), oligomycin A, protease inhibitor cocktail, peroxidase from horseradish, antimycin A (AntA), rotenone, pyruvate, tetramethyl-p-phenylenediamine (TMPD) and most other reagents were acquired from Sigma-Aldrich Co. (St. Louis, MO, USA). Ru360 was from Calbiochem, Merck Millipore. Blasticidin and Lipofectamine^®^ 3000 were obtained from Invitrogen (Paisley, UK). Fetal bovine serum (FBS), horse serum (HS) and OPTIMEM medium were purchased from GIBCO (Paisley, UK). Hoechst 33342 nucleic acid stain was purchased from Invitrogen/Molecular probes (Life Technologies Corporation, Carlsbad, CA, USA). Bovine serum albumin (BSA) was acquired from Santa Cruz Biotechnology (Santa Cruz Biotechnology, Inc., Santa Cruz, TX, USA). BioRad Protein Assay, polyvinylidene fluoride (PVDF) membrane and iQ SYBR Green Supermix were obtained from BioRad Laboratories, Inc. (Hercules, CA, USA). ECF substrate was purchased from GE Healthcare (GE Healthcare Bio-Sciences, Pittsburgh, PA, USA). XF24 cell culture microplates were purchased from Seahorse Bioscience (Billerica, MA, USA). Fura-2/AM, Rhodamine 123 (Rh123) and Amplex^®^Red probes were obtained from Molecular Probes/Invitrogen (Eugene, OR, USA). Ataxin-3 MAB5360 antibody (1:1000) was from Chemicon; TFAM ab131607 (1:500), p(Ser65)-parkin Ab154995 (1:500) and parkin Ab15954 (1:1000) antibodies were from Abcam; Mfn2 M6319 (1:1000) and β-actin A5316 (1:50,000) antibodies were from Sigma; Cx II (70 kDa subunit) A11142 antibody (1:10,000) was from Molecular Probes; Drp-1 #611112 (1:500) and cytochrome *c* #556433 (1:500) antibodies were from BD Biosciences; PGC1-α(K15) antibody sc-5816 (1:300) was from Santa Cruz Biotechnology; and polyglutamine (IC2) antibody MAB1574 (1:1000) was from Merck Millipore. NZYol and NZY First-Strand cDNA Synthesis Kits were purchased from NZYTech (Lisbon, Portugal). Puregene tissue kit was obtained from Qiagen (Hilden, Germany).

### 2.2. Methods

#### 2.2.1. Cell Culture

PC6-3 cell lines expressing wild-type (Q28) or expanded (Q108) human ataxin-3 were obtained from Dr. Henry L. Paulson, Department of Neurology, University of Michigan, MI, USA. The PC6-3 cell line is a subline of the immortalized PC12 cell line, which is obtained from rat adrenal gland pheochromocytoma [18]. Cells were maintained in RPMI medium supplemented with 10% (*v*/*v*) inactivated house serum (HS), 5% (*v*/*v*) inactivated fetal bovine serum (FBS), 1% (*v*/*v*) streptomycin/penicillin (100 units/mL of penicillin plus 100 μg/mL of streptomycin), 100 μg/mL of hygromycin and 2.2 μg/mL of blasticidin. The expression of ATXN3 was regulated through the addition of 1 μg/mL of doxycycline to the culture medium for 48 h before experiments. Cells were kept in uncoated T75 flasks, in an upright position, using an incubator chamber containing 95% air, 5% CO_2_ and 100% humidity at 37 °C.

#### 2.2.2. Animals

CMVMJD135 (MJD135, n = 9, CAG repeat: 140 ± 2) and wild-type littermates (WT, *n* = 8), with 24-week-old male mice, under a C57Bl/6 background, were used. The MJD135 mouse colony has been previously described [19] and expresses an expanded version of the human ATXN3c cDNA (the 3 UIMs containing a variant of ataxin-3) under the regulation of the CMV promoter (ubiquitous expression) at near-endogenous levels and manifests MJD-like motor symptoms that appear gradually and progress over time, being fully symptomatic at the selected age [19,20]. All animals (specific pathogen-free health status) were maintained under standard laboratory conditions: an artificial 12 h light/dark cycle (lights on from 8:00 to 20:00 h), with an ambient temperature of 21 ± 1 °C and a relative humidity of 50–60%. All animal procedures were performed to minimize exposure to stress and animal suffering, in accordance with the European Union Directive 2010/63/EU. Health monitoring was performed according to the Federation of European Laboratory Animal Science Associations (FELASA) guidelines. The specified pathogen-free health status was confirmed by monitoring sentinel mice maintained in the same animal housing room. Before the experiments, the animals were weighted and euthanized via decapitation, and their brains were then harvested and weighted.

#### 2.2.3. Isolation of Neuronal and Non-Neuronal Mitochondria

After being macrodissected, the cerebellum and brainstem were immediately subjected to mitochondrial isolation using discontinuous Percoll density gradient centrifugation, according to [21]. Briefly, the cerebellum and brainstem were homogenized in an ice-cold isolation buffer (225 mM of mannitol, 75 mM of sucrose, 5 mM of HEPES-KOH, 1 mM of EGTA, pH 7.2) and further centrifuged at 1100× *g* at 4 °C for 2 min. The supernatant was submitted to Percoll gradient centrifugation, and the pellet containing mitochondria was washed once with a washing buffer (250 mM of sucrose, 5 mM of HEPES-KOH, 0.1 mM of EGTA, pH 7.2) and further centrifuged at 10,000× *g* for 5 min at 4 °C. The final pellet containing isolated mitochondria was resuspended in the washing buffer, and protein was quantified using the BioRad protein assay. Mitochondrial fractions were immediately used for measurement of oxygen consumption via Seahorse analysis, mitochondrial membrane potential, mitochondrial calcium (Ca^2+^) handling and hydrogen peroxide (H_2_O_2_) production, or kept at −80 °C for later use.

#### 2.2.4. Sample Preparation and Western Blotting

##### Total Fractions

PC6-3 cells were centrifuged at 800× *g* for 5 min at 4 °C and washed in an ice-cold phosphate-buffered saline (PBS) solution (137 mM of NaCl, 2.7 mM of KCl, 1.4 mM of K_2_HPO_4_, 4.3 mM of KH_2_PO_4_, pH 7.4). These cells were resuspended in ice-cold RIPA buffer (50 mM of TRIS-HCl, 5 mM of EGTA, 150 mM of NaCl, 0.5% DOC, 0.1% SDS, 1% Triton X-100, pH 7.4), supplemented with 1 mM of sodium orthovanadate, 1 mM of phenylmethylsulphonyl fluoride (PMSF), 180 nM of okadaic acid, 50 mM of sodium fluoride (NaF), 1 mM of dithiothreitol (DTT) and 1 μg/mL of protease inhibitor cocktail (chymostatin, pepstatin A, leupeptin and antipain). The homogenates were sequentially frozen and thawed in liquid nitrogen three times and centrifuged at 20,800× *g* at 4 °C for 10 min. The resulting supernatants were collected and stored for later use.

##### Mitochondrial and Cytoplasmic-Enriched Fractions

Cells were centrifuged at 800× *g* at 4 °C for 5 min and washed in ice-cold PBS. These cells were then resuspended in 500 μL of ice-cold sucrose buffer (250 mM of sucrose, 20 mM of HEPES, 10 mM of KCl, 1.5 mM of MgCl_2_, 1 mM of EDTA, 1 mM of EGTA, pH 7.4), supplemented as described in the above section, and further homogenized with 40 strokes using a Potter-Elvejhem 377 homogenizer with a Teflon pestle at 280 rpm and then centrifuged at 1300× *g* at 4 °C for 12 min to pellet the nuclei and cell debris. The obtained supernatant was centrifuged at 11,900× *g* at 4 °C for 20 min, and the pellet containing the mitochondrial-enriched fraction was resuspended in the sucrose buffer. The supernatant (cytoplasmic fraction) was further subjected to protein precipitation using 15% trichloroacetic acid (TCA), and the extracts were centrifuged at 16,300× *g* at 4 °C for 10 min. The cytoplasmic-enriched pellet was resuspended in the sucrose buffer and pH was adjusted to 7.0 with 10 M of KOH.

##### Preparation of Mitochondrial Extracts from Isolated Mitochondria

Freshly or frozen isolated mitochondria from the cerebellum and brainstem were resuspended in supplemented ice-cold RIPA buffer (1:1). Each sample was then subjected to sonication three times (5–10 s/pulse) and centrifuged for 10 min at 20,800× *g* at 4 °C. The supernatant containing soluble mitochondrial proteins was collected and kept for further analysis.

##### Western Blotting

Equivalent amounts of protein were denatured with a denaturing buffer (50 mM of Tris-HCl, pH 6.8, 5% glycerol, 2% SDS, 600 mM of DTT and 0.01% bromophenol blue) at 95 °C for 5 min. Protein separation was performed using 7.5–15% SDS-PAGE and electroblotted onto PVDF membranes. The membranes were blocked for 1 h at room temperature in 5% (*w*/*v*) BSA in Tris-buffered saline (25 mM of Tris-HCl, pH 7.6, 150 mM of NaCl) with 0.1% Tween-20 (TBS-T), followed by overnight incubation with the primary antibodies described above at 4 °C. These membranes were washed with TBS-T 3 times for 15 min and then incubated with the secondary antibodies for 1 h at room temperature. All antibodies were prepared in 5% (*w*/*v*) BSA in TBS-T. Immunoreactive bands were visualized after incubation with ECF substrate using the ChemiDoc Touch Imaging System (Bio-Rad, Hercules, CA, USA). The bands were quantified using the Image Lab software (Bio-Rad).

#### 2.2.5. Measurement of O_2_ Consumption Using Seahorse Analyzer

Freshly isolated mitochondria (5 µg) from the cerebellum and brainstem of MJD135 and WT mice were resuspended in an ice-cold mitochondrial assay solution (MAS: 70 mM of sucrose, 220 mM of mannitol, 10 mM of KH_2_PO_4_, 5 mM of MgCl_2_, 2 mM of HEPES, 1 mM of EGTA and 0.2% (*w*/*v*) fatty acid-free BSA) containing 10 mM of succinate (Cx II substrate) and 2 μM of rotenone (Cx I inhibitor) for coupled oxygen consumption; in a second protocol, mitochondria were resuspended under uncoupled conditions by using MAS containing 10 mM of pyruvate, 2 mM of malate (mitochondrial substrates) and 4 μM of FCCP (mitochondrial uncoupler); mitochondria were allowed to adhere to cell culture XF24 microplates pre-coated with polyethyleneimine (PEI, 1:15,000 dilution from a 50% solution) via centrifugation at 4000× *g* for 20 min at 4 °C, as previously described [21]. The multiwell plate containing 5 μg of adherent mitochondria per well was incubated for 8 min at 37 °C in a non-CO_2_ incubator. Mitochondria respiration was assessed under coupled conditions via sequential addition of 4 mM of ADP, 2.5 μg/mL of oligomycin (Oligo, ATP synthase inhibitor), 4 μM of FCCP and 4 μM of AntA (AntA, Cx III inhibitor). In the second protocol, the activity of mitochondrial complexes (Cx) was assessed via sequential addition of 2 μM of rotenone (Rot), 10 mM of succinate (Suc), 4 μM of AntA and 10 mM of ascorbate/100 μM of TMPD (Asc/TMPD, for reduction of cytochrome *c*) under uncoupled conditions, as described above. Oxygen consumption rate (OCR) was measured at three consecutive time points before and after the injection of each drug using a Seahorse XF24 flux analyzer (Seahorse Bioscience, Billerica, MA, USA). The respective OCR described in the results were calculated according to [21].

O_2_ consumption by PC6-3 cells, cultured as described before, was also measured by using the XF24 flux analyzer, as described [20]. PC6-3 cells were grown in poly-D-lysine precoated XF24 microplates 48 h before the experiment at a density of 5000 cells per well. On the day of experiments, the cell culture medium was removed, and cells were washed two times with 1 mL of DMEM5030 medium, pH 7.4, and supplemented with 2 g/L of glucose and 0.3 g/L of glutamine, to fully remove the previous medium. Then, these cells were incubated in 450 µL of DMEM at 37 °C in a CO_2_-free incubator for 1 h. Sequential injection of mitochondrial inhibitors, oligomycin (2.5 μg/mL), FCCP (4 μM) and AntA (4 μM) plus rotenone (2 μM), was performed to evaluate basal respiratory capacity, maximal respiration in the presence of FCCP, oligomycin-sensitive O_2_ consumption coupled to ATP synthesis and proton (H^+^) leak (H^+^ leak). The results are expressed in picomoles of O_2_ per minute per microgram of protein (pmol O_2_/min/µg protein) and calculated as described in [20].

Glycolysis was also analyzed by using the XF24 flux analyzer to measure the extracellular acidification rate (ECAR, mpH/min) of the surrounding cell medium. For the ECAR experiments, DMEM5030 medium, pH 7.4, was supplemented with 0.3 g/L glutamine, and the cells were subjected to sequential addition of glucose (10 mM), oligomycin (2.5 μg/mL) and 2-DOG (10 mM). Glycolytic capacity was calculated according to [22]. At the end of the experiment, the protein level was determined for each well to correct for the amount of protein per well, and the results are expressed per µg protein.

#### 2.2.6. Measurement of Total Levels of Adenine Nucleotides

The cerebellum or brainstem tissues were subjected to acidic extraction using 0.6 M perchloric acid. The extracts were then centrifuged at 20,800× *g* for 2 min at 4 °C to remove cell debris; the resulting pellet was solubilized in 1 M of NaOH and further analyzed for protein content using the Bio-Rad Protein assay. After neutralization with 3 M of KOH/1.5 M of Tris, the samples were centrifuged at 20,800× *g* for 5 min at 4 °C. The resulting supernatants were assayed for ATP, ADP and AMP determination via separation using reverse-phase high-performance liquid chromatography (HPLC), as described previously [23]. The chromatographic apparatus used was a Beckman System Gold controlled by a computer. The detection wavelength was 254 nm, and the column used was a Lichrospher 100 RP-18. An isocratic elution with 100 mM of phosphate buffer (KH_2_PO_4_), at pH 6.5, and 1% methanol was performed with a flow rate of 1 mL/min. Peak identity was determined by following the retention time of the standards: 2.213 min for ATP, 2.589 min for ADP and 3.560 min for AMP. The energy charge, defined as the ratio of the complete adenylate pool, was calculated as [[ATP + (0.5 ADP)]/(AMP + ADP + ATP)].

#### 2.2.7. Mitochondrial Membrane Potential

Mitochondrial membrane potential (ΔΨ_m_) was assessed using the fluorescent probe Rhodamine 123 (Rh123), which predominantly accumulates in polarized mitochondria accordingly to [21]. Briefly, 10 μg of freshly isolated mitochondria from both the cerebellum and brainstem were resuspended in a KCl-reaction buffer containing 125 mM of KCl, 3 mM of K_2_HPO_4_, 0.5 mM of MgCl_2_, 10 mM of HEPES and 10 μM of EGTA, at pH 7.4, plus 50 nM of Rh123, 0.1 mM of ADP and supplemented with 3 mM of succinate plus 3 mM of glutamate, to feed mitochondrial Cx II, and basal fluorescence was immediately recorded. Succinate was used in combination with glutamate to prevent the accumulation of oxaloacetate and inhibition of succinate dehydrogenase, as previously described (e.g., [24]). For ΔΨ_m_ determination in PC6-3 cells, the samples containing 0.5 × 10^6^ cells were incubated in Krebs buffer containing 132 mM of NaCl, 4 mM of KCl, 1 mM of CaCl_2_, 1.2 mM of NaH_2_PO_4_, 1.4 mM of MgCl_2_, 6 mM of glucose and 10 mM of HEPES (pH 7.4), plus 3 μM of Rh123, for 30 min at 37 °C. In both experiments, fluorescence (505 nm excitation and 525 nm emission) was measured for 5 min (basal), followed by the addition of 2.5 µM of FCCP plus 2.5 µg/mL of oligomycin, which produced maximal mitochondrial depolarization. Fluorescence was measured at 30 °C for isolated mitochondria and at 37 °C for PC6-3 cells using a microplate reader, Spectrofluorometer Gemini EM (Molecular Devices, San Jose, CA, USA).

#### 2.2.8. Mitochondrial Ca^2+^ Uptake

Mitochondrial calcium uptake was assessed in the mitochondria isolated from the cerebellum and brainstem using the fluorescence probe Calcium Green-5N (Ca^2+^ Green), which binds extramitochondrial calcium [21]. Calcium Green-5N fluorescence was measured at 30 °C using a microplate reader, Spectrofluorometer Gemini EM (Molecular Devices, San Jose, CA, USA), at 506 nm excitation and 532 nm emission. For this purpose, 5 μg of isolated mitochondria was resuspended in a KCl-reaction buffer (described in the previous section) containing 150 nM of Ca^2+^ Green plus 0.1 mM of ADP and 1 μM of oligomycin, supplemented with succinate (3 mM) plus glutamate (3 mM), as described above. After basal fluorescence recording, mitochondria were subjected to two sequential loads of 10 μM of Ca^2^^+^. A third load of 2 μM of FCCP was also performed to assess calcium release from mitochondria and, thus, mitochondrial calcium retention. Extramitochondrial Ca^2+^ (area under the curve) was quantified for the second Ca^2+^ pulse. The effect of 10 μM of RU360, a mitochondrial calcium uniporter (MCU) inhibitor, was also tested.

#### 2.2.9. Intracellular Ca^2+^ Recordings

The levels of cytosolic free calcium in PC6-3 cells were measured using the fluorescent probe Fura-2/AM, which permeates the plasma membrane and has a high affinity for calcium. Cells (0.5 × 10^6^ cells) were incubated in Krebs buffer containing 5 μM of Fura-2/AM at 37 °C for 30 min. These cells were then centrifuged at 70× *g* for 5 min, and the pellet was washed once in Krebs buffer. Basal fluorescence was measured at 37 °C using a microplate reader, Spectrofluorometer Gemini EM (Molecular Devices, San Jose, CA, USA), with 340/380 nm excitation and 510 nm emission wavelengths. After the baseline recording, the cells were subjected with the addition of 2.5 µM of FCCP plus 2.5 µg/mL of oligomycin to depolarize mitochondria. The levels of intracellular free calcium were calculated as the ratio of the fluorescence intensities at 340 nm and 380 nm, corresponding to maximal fluorescence of the probe in the presence of calcium and in the absence of calcium, respectively.

#### 2.2.10. H_2_O_2_ Levels

The levels of mitochondrial H_2_O_2_ were measured using the Amplex^®^Red hydrogen peroxide/peroxidase method in isolated mitochondria and in PC6-3 cells. The reagent Amplex^®^Red (10-acetyl-3.7-dihydroxyphenoxazine), in the presence of the enzyme horseradish peroxidase (HRP), reacts with H_2_O_2_ to form the fluorescent product resorufin; in this way, the fluorescence intensity is proportional to the amount of H_2_O_2_. KCl-reaction buffer (described above) plus 2 μM of Amplex^®^Red and 0.5 units HRP, supplemented with 3 mM of succinate plus 3 mM of glutamate as previously described, was subjected to basal fluorescence determination (571 nm excitation and 585 nm emission), and then, 5 µL of freshly isolated mitochondria (5 μg protein) was added. H_2_O_2_ levels were also measured in 0.5 × 10^6^ PC6-3 cells in Krebs buffer containing 2 μM of Amplex^®^Red and 0.5 units HRP. Fluorescence traces were obtained up to 10 min at 30 °C using a microplate reader, Spectrofluorometer Gemini EM (Molecular Devices, San Jose, CA, USA).

#### 2.2.11. Analysis of mRNA Levels

Gene expression was evaluated using reverse transcription quantitative real-time PCR (RT-qPCR). Total RNA was extracted from MJD135 and WT mouse cerebellum and brainstem total extracts using NZYol, according to the instructions of the supplier. RNA concentration was determined using a NanoDrop 2000c spectrophotometer (Thermo Scientific, Waltham, MA, USA). Complementary DNA (cDNA) was then synthesized from 500 ng of total extracted RNA using the NZY First-Strand cDNA Synthesis Kit, following the manufacturer’s instructions. The PCR reactions were performed in 10 μL volumes containing 5 μL of iQ SYBR Green Supermix (BioRad, Hercules, CA, USA), 300 nM of each primer (as described below) and 50 ng of cDNA template in a Bio Rad CFX96 Real-Time PCR Detection System using the following cycling conditions: initial denaturation at 95 °C for 3 min, followed by 40 cycles of denaturation at 95 °C for 15 s and annealing/extension at 55–61.7 °C for 45 s. At the end, the samples were subjected to a melting curve analysis to confirm the absence of unspecific amplification products and primers dimers. Samples containing no template were included as negative controls in all experiments. The reactions were run in duplicates and analysis of gene expression was performed using the ΔΔCT method. β-2M was used as an internal control for all samples. The PCR primer sequences used were as follows (forward primer (F); reverse primer (R)):

*Cyt c*—F: CCAAATCTCCACGGTCTGTTC; R: ATCAGGGTATCCTCTCCCCAG.
*Ucp2*—F: AGCCCACGGATGTGGTAAAG; R: CTCTCGGGCAATGGTCTTGT.
*Ucp4*—F: CCTGGACACCTCCAATCCAC; R: TCCTGACCTGACCTCTCTCG.
*Ucp5*—F: GTAAGCGGACATCAGAAAAGTTCC; R: GCCGAACTCGGCAACAATAG.
*β-2M*—F: CCTTCAGCAAGGACTGGTCT; R: TCTCGATCCCAGTAGACGGT.

#### 2.2.12. Transmission Electron Microscopy (TEM) Analysis

For mitochondrial morphometric characterization using TEM, the mice were anesthetized with a mixture of ketamine (75 mg/kg, Imalgène 1000, Merial, USA) and medetomidine (1 mg/kg, Dorbene Vet, Pfizer, USA) via intraperitoneal injection and transcardially perfused with 0.9% saline solution followed by 4% paraformaldehyde for tissue fixation. The cerebellum was macrodissected and stored in a solution containing 2% paraformaldehyde and 2% glutaraldehyde (G5882, Sigma-Aldrich, Darmstadt, Germany) in 0.1 M of sodium cacodylate (C0250, Sigma-Aldrich) at pH 7.4. The specimens were processed as described in [25]. Ultrathin sections (approximately 60 nm thick) containing the cerebellar cortex were then observed under a JEOL JEM-1400 transmission electron microscope (Zeiss). On average, 65 nonoverlapping images were acquired at 30,000× magnification (except a few images that were acquired at 20,000 or 40,000×; whenever this occurred, the obtained values were adjusted accordingly), per animal (MJD135, *n* = 5; WT, *n* = 4). Mitochondrial morphometric analysis was carried out using the FIJI software [26]. To avoid potential biases, the experimenter was blind to the genotype. Mitochondria with a discernible double bi-lipidic layer were manually traced using the freehand tool.

Several shape descriptors and size measurements of each mitochondrion were retrieved and evaluated, namely area (μm^2^), perimeter (μm), aspect ratio (major axis/minor axis) and minimum Feret’s diameter (μm). Mitochondrial sphericity was evaluated based on circularity [4π × (area/perimeter^2^)] and roundness [4 × (area)/(π × major axis^2^)] indexes, where values near 1 denote a perfect circular shape. On average, approximately 1000 mitochondria per animal were evaluated.

#### 2.2.13. Analysis of mtDNA Copy Number

Total genomic DNA was extracted from 34-week old MJD135 and WT littermate mice’s cerebellum and brainstem using Puregene^®^ Core kit A (Qiagen), according to the instructions of the supplier. DNA concentration was determined using a NanoDrop 2000c spectrophotometer (Thermo Scientific, Waltham, MA, USA). Copy number of mtDNA was determined by carrying out qPCR in a total volume of 10 μL, containing 5 μL of iQ SYBR Green Supermix, 400 nM of each primer and 20 ng of template DNA. The reactions were performed in a BioRad CFX96 Real-Time PCR Detection System using the following protocol: pre-incubation at 95 °C for 3 min, followed by 40 cycles of denaturation at 95 °C for 15 s and annealing/extension at 60 °C for 45 s. At the end, the samples were subjected to a melting curve analysis, and the specificity of the primers (one PCR product amplified) was confirmed as a single melt peak. The ΔΔCT method was used in the calculations of fold changes. Cytosolic carboxypeptidase 2 (CCP2) was used as a nuclear genome control for normalizing mtDNA levels. The PCR primer sequences used were as follows (forward primer (F); reverse primer (R)):

*mtDNA*—F: CGACCTCGATGTTGGATCA; R: AGAGGATTTGAACCTCTGG.
*CCP2*—F: CCAAATCTCCACGGTCTGTTC; R: ATCAGGGTATCCTCTCCCCAG.

#### 2.2.14. Statistical Analysis

All graphs and statistical analysis were performed using GraphPad Prism 8 (GraphPad Software, San Diego, CA, USA). The data are expressed as mean ± SEM of the number of experiments, as described in the figure legends. Comparisons between two groups were analyzed using Mann–Whitney test or Student’s *t*-test, as indicated in the figure legends. Significance was accepted at *p* < 0.05. Sample normality was tested using Shapiro–Wilk test.

## 3. Results

### 3.1. MJD135 Mice Exhibit Decreased Cerebellar Mitochondrial Respiration and Reduced ATP Production

At 24 weeks old, transgenic CMVMJD135 mice (hereon referred to as MJD135 mice) show a fully developed motor phenotype, as well as spinal cord and brain pathology, as evaluated previously by us [20]. In the present study, a significant decrease in body weight by about 17% (Figure S1A) and a significant gross brain atrophy by about 6% (Figure S1B) were observed in the MJD135 animals, when compared to the WT littermate mice. The expression of mATXN3 in the MJD models used in this work was also evaluated via Western blotting (using antibodies against ATXN3 and polyQ expansions higher than 37 glutamines; Figures S1C,D, S5 and S6A). Our results show the presence of mATXN3 in the total extracts obtained from the cerebellum and brainstem (Figure S1C, two brain regions mostly affected in MJD) of MJD135 mice; however, no labeling of mATXN3 or polyglutamine expansion was observed in the mitochondria isolated from these two brain areas (data not shown), probably as a result of a labile interaction of the protein with the organelle and/or detachment due to the isolation procedure based on Percoll density gradient using BSA, as suggested for displacement of mHTT from its binding sites on mitochondria (e.g., [27]). 

**Figure 1:**
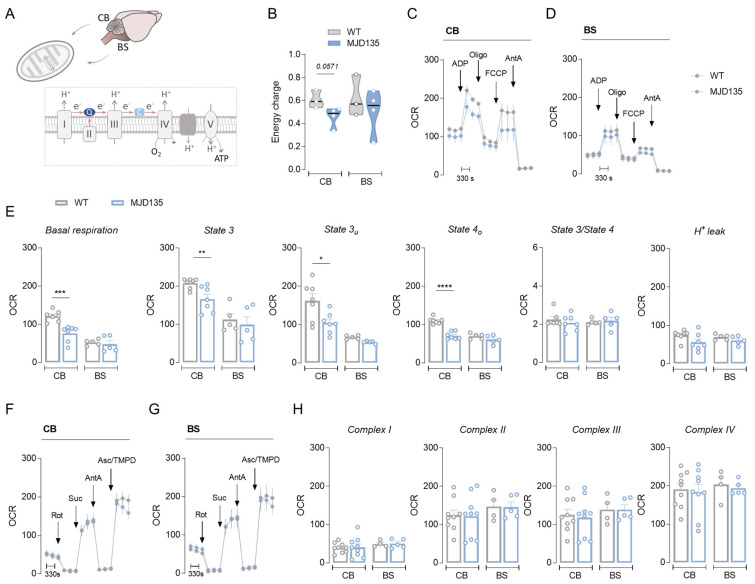
Oxygen consumption rates (OCR) in MJD135 mouse cerebellar and brainstem mitochondria. Mitochondrial respiration was measured in cerebellar and brainstem isolated mitochondria (**A**) using a Seahorse flux analyzer, and OCR is expressed in pmol/min/μg protein. The levels of adenine nucleotides and energy charge (**B**) were determined in the total extracts from the cerebellum and brainstem of 24-week-old MJD135 and WT mice as described in the Methods section. Levels of respiratory coupling were analyzed using MAS containing 10 mM of succinate plus 2 μM of rotenone under sequentially injection of mitochondrial inhibitors and substrates (final concentration: 4 mM of ADP, 2.5 μg/mL of oligomycin [Oligo], 4 μM of FCCP and 4 μM of antimycin A [AntA]), as shown in the representative traces for cerebellum (CB; **C**) or brainstem (BS; **D**). Basal respiration or state 2, state 3 (ADP-induced respiration), state 3_u_ (maximal respiration), state 4_o_ (ATP production), state3/state4 and H^+^ leak values were calculated for both regions as described in the Methods section (**E**). The activity of mitochondrial respiratory chain complexes was analyzed using MAS supplemented with 10 mM of pyruvate, 2 mM of malate and 4 μM of FCCP. Mitochondrial inhibitors and substrates were then sequentially injected (final concentration: 2 μM of rotenone, 10 mM of succinate, 4 μM of antimycin A and 10 mM of ascorbate/ 100 μM of TMPD), as shown in the representative traces for CB (**F**) or BS (**G**), and Cx I-IV activities for both regions were calculated as described in the Methods section (**H**). Data are expressed as the mean ± SEM of the experiments performed in independent mitochondrial preparations obtained from 3 to 7 mice from each genotype, run in duplicate or triplicate. Statistical analysis: * *p* < 0.05, ** *p* < 0.01, *** *p* < 0.001, and **** *p* < 0.0001 when compared with WT mitochondria based on Mann–Whitney test or Student’s *t*-test.

Mitochondrial respiration was analyzed using a Seahorse XF24 flux analyzer in functional isolated mitochondria obtained from the cerebellum and brainstem of 24-week-old MJD135 and WT mice (Figure 1A). Cerebellar, but not brainstem, mitochondria from MJD135 mice exhibited a decrease in mitochondrial respiration in basal conditions (state 2, mitochondrial respiration in the presence of substrates), after being full energized with ADP (state 3 respiration), and maximal respiration (state 3_u_ respiration, achieved after FCCP stimulus in order to completely depolarize the organelle). In addition, there was a decrease in mitochondrial ATP production (state 4 respiration, evaluated after addition of oligomycin), although no significant differences in state3/state4 ratio (or respiratory control ratio, an index of how coupled respiration is to phosphorylating ADP) or in H^+^ leak were observed, when compared to WT mice (Figure 1C–E). H^+^ leak is defined by a process by which protons return to the mitochondrial matrix independently of ATP synthase (e.g., [28]). In accordance with unchanged H^+^ leak, unaltered mRNA levels of *Ucp2*, *Ucp4* and *Ucp5* (the latter two are highly abundant in mouse brain [29]) were observed in both brain areas from mutant versus WT mice (Figure S2). Interestingly, decreased mitochondrial ATP generation was corroborated by a tendency for a reduced energy charge in the total fractions of the cerebellum from MJD135 mice (Figure 1B, *p* = 0.0571). However, no significant changes in the activities of mitochondrial respiratory chain Cx I, II, III and IV were observed in either brain region (Figure 1F–H).

### 3.2. PC6-3 Q108 Cells Show Decreased Mitochondrial Respiration and ATP Production and Enhanced Glycolysis Capacity

The presence of mATXN3 in the mitochondrial fractions derived from PC6-3 cells expressing mutant (Q108) versus wild-type (Q28) human ATXN3, as well as in total and cytoplasmic extracts, shows that expanded/mATXN3 is able to interact with mitochondria (Figures S1D and S6A). The detection of mATXN3 in the mitochondrial fractions from PC6-3 Q108 cells obtained in this work was in accordance with previous studies, in which two small ATXN3 isoforms (29- and 49 kDa) were found in mitochondrial-enriched fraction (P2) obtained via subcellular fractionation of Hela cells [30].

**Figure 2:**
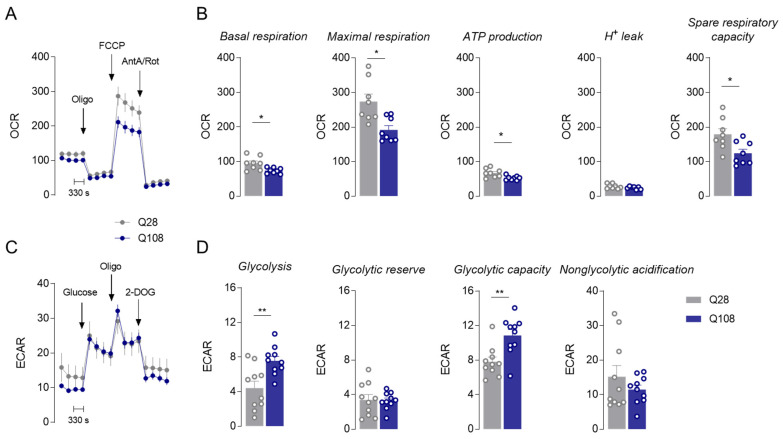
Mitochondrial respiration in PC6-3 cells. Mitochondrial respiration was measured in Q108 and Q28 PC6-3 cells using a Seahorse flux analyzer, and OCR is expressed in pmol/min/μg protein. Levels of respiratory coupling were analyzed in DMEM medium under sequentially injection of 2.5 μg/mL of oligomycin (Oligo), 4 μM of FCCP and 4 μM/2 μM of antimycin A (AntA)/rotenone (Rot), as shown in the representative traces for Q28 or Q108 (**A**). Basal respiration, maximal respiration, ATP production, H^+^ leak and spare respiratory capacity (**B**) were calculated for both cells as described in the Methods section. Extracellular acidification rate (ECAR; mpH/min/μg protein) was evaluated in Q108 and Q28 PC6-3 cells and assessed via sequential addition of 10 mM of glucose, 2.5 μg/mL of oligomycin and 10 mM of 2-deoxy-D-glucose (2-DOG), as shown in the representative traces (**C**). Glycolysis, glycolytic reserve, glycolytic capacity and nonglycolytic acidification were calculated as described in the Methods section (**D**). Data are expressed as the mean ± SEM of 8–10 experiments run in duplicate or triplicate and performed in independent cell preparations. Statistical analysis: * *p* < 0.05 and ** *p* < 0.01 when compared with Q28 cells based on Mann–Whitney test or Student’s *t*-test.

Oxygen consumption rate measurements show a significant decrease in both basal and maximal respiration, along with decreased ATP synthesis and spare respiratory capacity and unchanged H^+^ leak in PC6-3 Q108 cells compared to Q28 cells (Figure 2A,B), suggesting compromised mitochondrial function in mutant PC6-3 Q108 cells. Moreover, under these conditions, mutant PC6-3 cells show enhanced glycolysis and glycolytic capacity, potentially acting as compensatory mechanism, but unaltered glycolytic reserve or nonglycolytic acidification, as shown in Figure 2C,D.

### 3.3. MJD135 Mouse Cerebellum and PC6-3 Q108 Cells Display Decreased Mitochondrial Membrane Potential (ΔΨ_m_)

**Figure 3:**
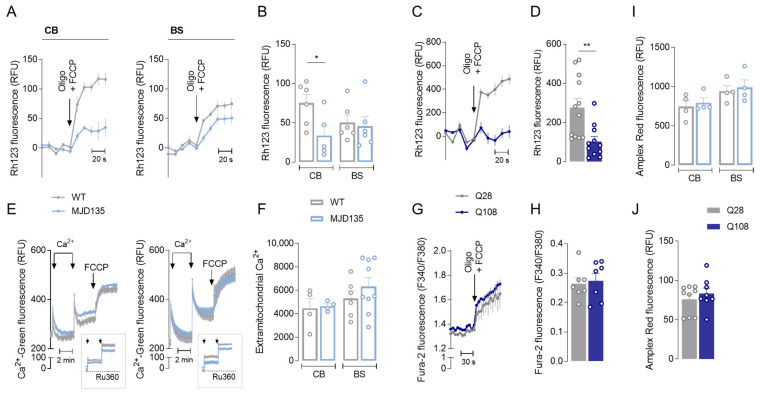
Mitochondrial membrane potential (ΔΨm), mitochondrial Ca^2+^ handling and ROS levels in MJD135 mouse cerebellar and brainstem mitochondria and in PC6-3 Q108 cells. ΔΨm was determined in cerebellar and brainstem mitochondria using the fluorescence probe Rhodamine 123 in a medium containing 0.1 mM of ADP and 3 mM of succinate, plus 3 mM of glutamate, and subjected to Oligo + FCCP full depolarization, as described in the Methods section. Traces are shown in (**A**) and quantification of ΔΨm for CB or BS is presented in (**B**). Q108 or Q28 PC6-3 cells were loaded with 3 μM of Rh123 in Krebs buffer and ΔΨm was evaluated under Oligo + FCCP full depolarization as described above. Traces are presented in (**C**) and quantification of ΔΨm is shown in (**D**). Mitochondrial Ca^2+^ handling was assessed using the fluorescence probe Calcium Green-5N (Ca^2+^ Green; 300 nM) in mitochondria isolated from CB and BS of MJD135 versus WT mice subjected to two loads of 10 μM of Ca^2+^. Then, a third load of FCCP (2 μM) was applied, as presented in traces. The insets represent the effect of MCU inhibitor, Ru360 (10 μM) (**E**). Extramitochondrial Ca^2+^ (area under the curve for the second pulse) was quantified as described in the Methods section (**F**). Mitochondrial Ca^2+^ was also evaluated in Q108 and Q28 PC6-3 cells loaded with Fura-2/AM and assessed as Ca^2+^ released by mitochondria and, thus, mitochondrial Ca^2+^ retention, under Oligo + FCCP full depolarization, as shown in traces (**G**), and quantification of mitochondrial Ca^2+^ content is shown in (**H**). Levels of H_2_O_2_ were also evaluated in CB or BS isolated mitochondria (**I**) or in Q108 and Q28 cells (**J**) using Amplex Red assay, as described in the Methods section. Data are expressed as mean ± SEM of mitochondria isolated from 4–6 mice from each genotype, run in quadruplicate, and 7–12 experiments in independent Q108 and Q28 PC6-3 cell lines, run in triplicate. Statistical analysis: * *p* < 0.05 and ** p < 0.01 when compared to the respective control based on Student’s *t*-test.

ΔΨ_m_ was assessed in the mitochondria from MJD135 mouse cerebellum and brainstem and PC6-3 cells expressing mATXN3. Our results show that cerebellar mitochondria from MJD135 mice exhibited a significant decrease in ΔΨ_m_, whereas no alterations were found in the mitochondria derived from the brainstem (Figure 3A,B). These results were corroborated by the cell model used in this work; indeed, PC6-3 Q108 cells also exhibited a significant decrease in ΔΨ_m_ (Figure 3C,D). These results suggest that the expression of mATXN3 may cause mitochondrial depolarization, particularly affecting cerebellar mitochondria, and may explain the decay in mitochondrial respiration without changes in individual complexes’ activity.

Because maintenance of ΔΨ_m_ is essential for normal mitochondrial functioning, mitochondrial calcium handling was further evaluated in the mitochondria isolated from MJD135 versus WT mouse cerebellum and brainstem. Both MJD135 cerebellar and brainstem mitochondria showed no significant alterations in Ca^2+^ uptake capacity in response to 10 μM of Ca^2+^ stimulus, when compared to WT mitochondria (Figure 3E,F). Under these conditions, mitochondria were able to release Ca^2+^ following FCCP-induced depolarization (Figure 3E). Ca^2+^ uptake was completely abolished in the presence of RU360 (10 μM), a selective inhibitor of the mitochondrial Ca^2+^ uniporter (MCU; Figure 3E inset), indicating that the uptake of Ca^2+^ by mitochondria was mediated by MCU. Similarly, mitochondrial calcium retention was also measured in PC6-3 cells by evaluating cytoplasmatic free Ca^2+^ under complete mitochondrial depolarization in the presence of oligomycin (ATP synthase inhibitor) plus FCCP (Figure 3G,H). Under these conditions, no significant differences in cytoplasmatic free Ca^2+^ were observed between PC6-3 Q108 and Q28 cells, suggesting unchanged mitochondrial Ca^2+^ retention under basal conditions despite the decrease in ΔΨ_m_ (Figure 3G,H). 

Considering that mitochondria, in particular Cx I and III, are the major producers of reactive oxygen species (ROS) [31], we also measured the levels of H_2_O_2_ in the mitochondria isolated from the cerebellum and brainstem of MJD135 versus WT mice, as well as in PC6-3 Q108 cells, which reflects H_2_O_2_ levels resulting from the equilibrium between its production and degradation. Our results show no major differences in basal mitochondrial H_2_O_2_ levels in both brain regions in mutant mice (Figure 3I) or in PC6-3 Q108 cells (Figure 3J), when compared to the control conditions.

Furthermore, no changes in the mitochondrial levels of Drp1 and Mfn2, which mediate mitochondrial fission and fusion, respectively, or mitochondrial morphology, as detected using TEM, were observed in the mitochondria isolated from MJD135 mouse cerebellum, when compared to WT mice (Figures S3 and S6B). In addition, we followed the changes in parkin phosphorylated at Ser65 (P(Ser65)-parkin) by PINK1 as a marker of PINK1/parkin mitophagy. No changes in P(Ser65)-parkin/parkin were observed in the mitochondria isolated from mutant mice or in the mitochondrial and cytosolic fractions derived from PC6-3 cells (Figure S3D,E), suggesting that the PINK1/parkin mitophagy pathway is not activated following the expression of mutant ATXN3.

### 3.4. Decreased Cytochrome c in Cerebellum of MJD135 Mice and PC6-3 Q108 Cells

**Figure 4:**
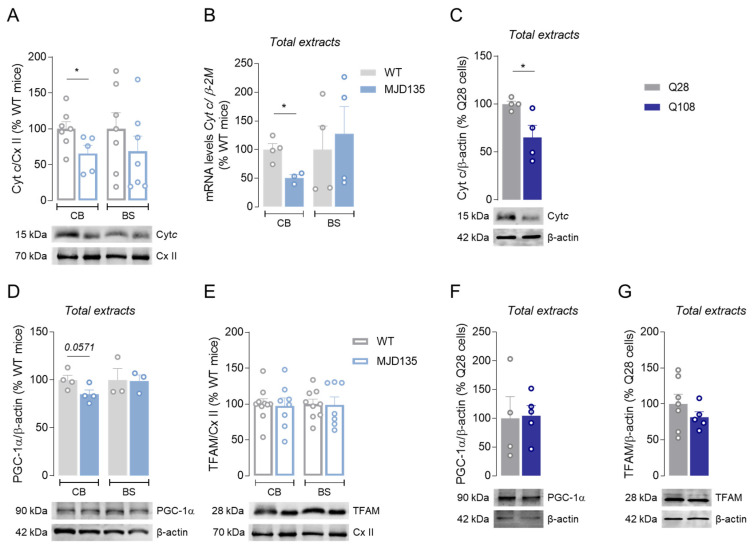
Levels of cytochrome *c*, PGC-1α and TFAM in MJD135 mouse cerebellar and brainstem mitochondria and in PC6-3 Q108 cells. Mitochondrial protein levels (**A**) and mRNA levels (**B**) of cytochrome *c* were evaluated in CB and BS of MJD135 versus WT mice using Western blotting and RT-PCR, respectively. Total protein levels of cytochrome *c* in PC6-3 Q108 and Q28 cells (**C**). Total protein levels of PGC1-α (**D**) and mitochondrial protein levels of TFAM (**E**) in CB and BS of MJD135 versus WT mice. Total protein levels of PGC1-α (**F**) and TFAM (**G**) in PC6-3 Q108 and Q28 cells. β-Actin or Cx II (70 kDa subunit) were used as the internal control loading for total or mitochondrial extracts, respectively. Data are expressed as the mean ± SEM of the experiments performed in independent mitochondrial preparations obtained from 3 to 7 mice from each genotype or 4–7 independent experiments using Q108 and Q28 PC6-3 cell lines. Statistical analysis: * *p* < 0.05 when compared to the respective control based on Student’s *t*-test.

Considering that cytochrome *c* is a key element of the mitochondrial respiratory chain, we also analyzed the protein levels of cytochrome *c* using Western blotting in cerebellar and brainstem isolated mitochondria (Figure 4A and Figure S4A), and the mRNA levels in total extracts from these two brain areas (Figure 4B). Interestingly, the cerebellum from MJD135 mice exhibited a significant decrease in cytochrome *c* protein levels in isolated mitochondria (Figure 4A) and *cytochrome c* mRNA levels in total extracts (Figure 4B), whilst no differences were observed in either cytochrome *c* protein or mRNA levels in the brainstem (Figure 4A,B). In addition, a significant decrease in cytochrome *c* protein levels was observed in the total extracts of the PC6-3 Q108 cell line compared to the Q28 cell line (Figure 4C and Figure S4B). Because cytochrome *c* promotes the transfer of electrons from Cx III to Cx IV, culminating with the production of ATP [32], our data are in accordance with the results of mitochondrial respiration obtained in MJD135 mouse cerebellar mitochondria and in PC6-3 Q108 cells.

Mitochondrial biogenesis is a multistep process that plays an important role in regulating the number of mitochondria in cells. To explain the changes in mitochondrial function in the cerebellum and brainstem of MJD135 mice and in PC6-3 Q108 cells, the levels of two proteins that play key roles in mitochondrial biogenesis, namely peroxisome proliferator-activated receptor γ coactivator-1 (PGC-1α) that directly regulates cytochrome *c* and mitochondrial transcription factor 1 (TFAM), were evaluated (Figure 4 and Figure S4C–F). Our results show no significant changes in PGC-1α levels in the cerebellum nor in the brainstem when analyzing MJD135 total extracts (Figure 4D and Figure S4C); concordantly, no significant changes were observed in the total extracts of PC6-3 Q108 cells when compared to the controls (Figure 4F and Figure S4E). Unaltered PGC-1α was accompanied by no changes in TFAM levels in both isolated mitochondria derived from the cerebellum and brainstem of MJD135 mice (Figure 4E and Figure S4D) or in mutant PC6-3 cells (Figure 4G and Figure S4F). Moreover, no significant differences were observed in the mtDNA/nDNA ratio in the cerebellum and brainstem of MJD135 mice (Figure S3F).

## 4. Discussion

The present study shows that mitochondrial dysfunction is a common feature in mouse brain and cellular models of MJD, namely in the cerebellum of MJD135 mice at symptomatic stage and in PC6-3 cells expressing expanded (Q108) human ATXN3. We provide evidence of compromised OCR parameters, which is in agreement with the trend of a decrease in energy charge in the cerebellum, but not in brainstem, largely suggesting a compromised cerebellar mitochondrial function in MJD135 mice. Our results also demonstrate decreased basal and maximal respiration, ATP synthesis and spare respiratory capacity, concomitantly with enhanced glycolytic capacity in PC6-3 Q108 cells, indicating impaired mitochondrial function in this cellular model of MJD. Enhanced glycolytic activity may act to counterbalance energy production under conditions of reduced mitochondrial ATP synthesis. Considering the importance of mitochondrial oxygen consumption for proper neuronal and brain activity, a compromised organelle function may underlie an increased susceptibility for neurodegeneration. Overall, the respiratory experiments in the MJD mouse model demonstrate that the cerebellum is more affected than the brainstem, which is in agreement with previous findings obtained by us showing that these mice exhibit higher accumulation of ATXN3 protein and mRNA levels in the cerebellum, followed by the brainstem, forebrain and spinal cord [19]. These data are in accordance with a recent study using SCA3 patient-derived fibroblasts, where reduced ATP production and dysregulation of parkin-VDAC1-mediated mitophagy were observed [17].

It was previously described that both normal and polyQ-expanded ATXN3 interact with components of the protein quality control system and mitochondria [13]. A decrease in the activity of mitochondrial Cx II was previously observed by us in nerve growth factor-differentiated PC6-3 Q108 cells [7]. Nevertheless, in the present work, no differences were observed in the activities of mitochondrial respiratory chain complexes (Cx I-IV) in the mitochondria isolated from both brain areas derived from MJD135 mice; this might be due to the differential levels of mATXN3 expression and/or interaction with mitochondria. Indeed, although we could not detect ATXN3 protein levels in isolated mitochondria in the present study, mitochondrial fractions isolated from undifferentiated PC6-3 Q108 cells showed detectable levels of mATXN3. In striatal mitochondria isolated from pre-/early symptomatic YAC128 mice, we also found retained mHTT interaction. In this HD study, mitochondrial deregulation was associated with enhanced respiratory chain activity and increased ROS levels, as observed in pre-manifest/prodromal versus manifest HD patients and in YAC128 transgenic mouse model at pre-/early symptomatic stage [33].

The co-transcriptional regulator PGC-1α is a key regulator of mitochondrial biogenesis, and has also been shown to play an important role in mitochondrial respiration, energy metabolism and cell viability [34,35,36]. Its role in ROS metabolism has also been highlighted as PGC-1α overexpression has been shown to be neuroprotective against oxidative stress [36,37]. In addition, the levels of PGC-1α and TFAM, the main transcriptional regulator of mitochondrial DNA (mtDNA) that coordinates the assembly of multiple DNA molecules and organizes mitochondrial chromatin, decrease in HD [38]. Accordingly, PGC-1α knockdown was shown to exacerbate the phenotype of HD mice, while its overexpression promoted neuroprotection [39,40]. Our results demonstrate unaltered PGC-1α levels in both MJD135 mouse cerebellar and PC6-3 Q108 cell total extracts. Additionally, cytochrome *c* protein and mRNA levels were significantly diminished in mouse cerebellum and in PC6-3 Q108 cells total extracts, indicating that despite the normal activity of Cx IV (measured under optimal substrate conditions), less cytochrome *c* is available to promote electron flow along the respiratory chain, resulting in decreased ATP production and respiration. Previous studies in models of neurodegenerative disorders showed reduced number of mitochondria and decreased levels of proteins involved in mitochondrial biogenesis [37,39,40]. However, we observed no alterations in PGC-1α or TFAM levels in both MJD135 mice and PC6-3 Q108 cells. This was concordant with the unaltered mtDNA/nDNA ratio in MJD135 mice. Of note, decreased mtDNA copy number and accumulation of mtDNA deletions were previously shown in another MJD mouse model of CMVMJD94 mice [11,12].

Many studies highlighted the importance of ΔΨm in mitochondrial function and, thus, in cell survival, providing the charge gradient for calcium handling and ROS production [41,42]. Earlier findings demonstrated that neurons expressing mATXN3 exhibited depolarized mitochondria, compromised Ca^2+^ handling and downregulation of genes involved in Ca^2+^ signaling [43,44,45]. Truncated mutant ATXN3 was shown to cause mitochondrial dysfunction, further leading to neurodegeneration in both neuroblastoma cells and in cerebellum of transgenic mice, suggesting that truncated mutant ATXN3 has a more severe effect than full-length mutant one [46]. Previous studies obtained by us in HD evidenced decreased ΔΨ_m_ which compromised their ability to regulate Ca^2+^ homeostasis in striatal neurons from YAC128 mice, thus contributing to neuronal dysfunction [46]. In addition, reduced respiratory profile and decreased ΔΨm in YAC128 mouse cortical and striatal neurons, and increased complexes activity along with H_2_O_2_ levels, in YAC128 mouse striatal mitochondria were also observed by us [47]. In this study, we showed that MJD135 mice exhibited decreased cerebellar, but not brainstem, ΔΨ_m_ without significant changes in mitochondrial Ca^2+^ handling capacity. Moreover, PC6-3 Q108 cells also displayed a significant decrease in ΔΨ_m_ and unchanged mitochondrial Ca^2+^ levels. Decreased ΔΨ_m_ in both MJD models is in accordance with reduced mitochondrial respiration and ATP production.

PGC-1α is part of a homeostatic cycle that is central to the control of ROS. As such, PGC-1α KO mice were found to present increased oxidative stress in the dopaminergic cells of the substantia nigra and hippocampal neurons [37]. In our study, unchanged ROS levels appear to correlate with maintained PGC-1α levels and activity of Cx I-IV.

Overall, our results are the first to suggest decreased cytochrome *c* levels in MJD (occurring independently of PGC-1α), which is translated into compromised mitochondrial function, particularly in the cerebellum. Additional studies may clarify the molecular pathways involved in these changes, as well as other possible mechanisms. Considering that mitochondrial dysfunction acts as a pathological mechanism in MJD, potential novel therapeutics might emerge for MJD.

## Data Availability

The datasets used and/or analyzed during the current study are available from the corresponding author upon reasonable request.

## Supplementary Materials

The following supporting information can be downloaded at https://www.mdpi.com/article/10.3390/cells12192397/s1, Figure S1: Total body and brain weight and analysis of mutant ATXN3 in MJD mice and PC6-3 cells; Figure S2: mRNA levels of *Ucp2/4/5* in mitochondrial or total extracts from cerebellum or brainstem; Figure S3: Mitochondrial Drp1, Mfn2 and p(Ser65)-parkin/parkin protein levels, TEM analysis and mtDNA/nDNA ratio in MJD versus control mice and PC6-3 cells; Figure S4: Full-length Western blots for PGC1-α, TFAM and cytochrome *c* in MJD135 mouse cerebellar and brainstem mitochondria and in PC6-3 cells; Figure S5: Full-length Western blots for mutant ATXN3 in MJD mice; Figure S6: Full-length Western blots for mutant ATXN3 in PC6-3 cells and mitochondrial Drp1 in MJD135 mouse cerebellum and brainstem.
