# Supplementary material for: Mitochondrial Dysfunction and Decreased Cytochrome c in Cell and Animal Models of Machado–Joseph Disease
Source: Cells. 2023 Oct 3;12(19):2397. doi: 10.3390/cells12192397 (PMC10571982; doi:10.3390/cells12192397)
Supplement: Supplementary file 1 [file cells-12-02397-s001.zip › cells-2448952-supplementary.pdf]

Supplementary Figures:

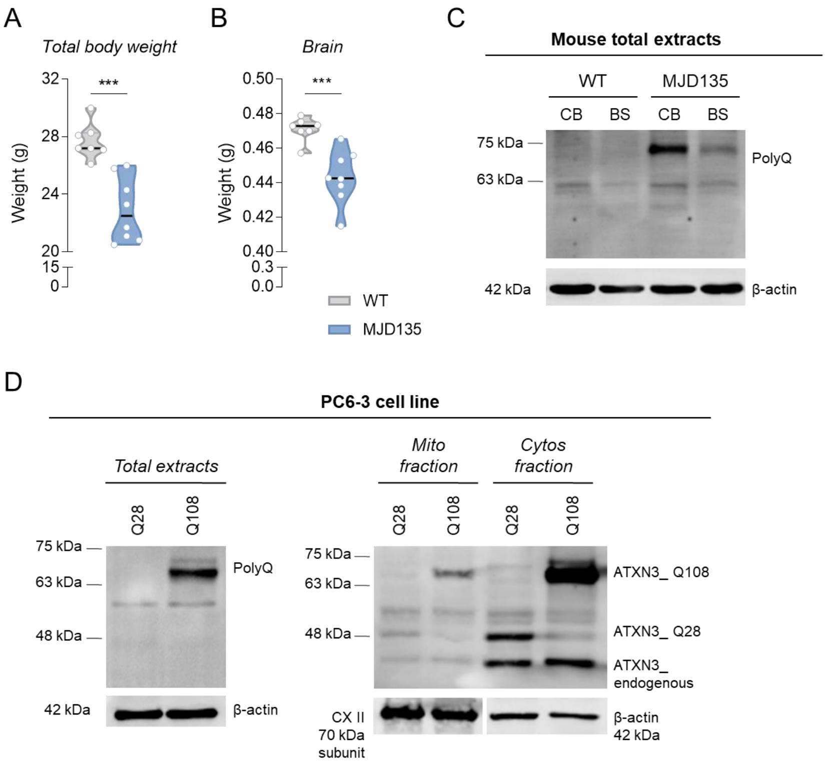

Figure S1. Total body and brain weight and analysis of mutant ATXN3 in MJD mice and PC6-3 cells. Total body (**A**), brain (**B**) weight were determined in 24-week-old MJD135 and WT mice. Total protein levels of polyglutamine expansion (polyQ) were assessed in cerebellum (CB) and brainstem (BS) of MJD135 versus WT mice by western blotting (**C**). ATXN3 levels were also evaluated in total extracts or mitochondrial and cytoplasmic fractions from PC6-3 Q108 versus Q28 cells (**D**). β-actin or Cx II (70 kDa subunit) were used as internal control loading for total/cytoplasmic or mitochondrial extracts, respectively. Data are the mean ± SEM of 7-8 mice from each genotype. Statistical analysis: ***p<0.001, compared to WT mice by Student's t-test.

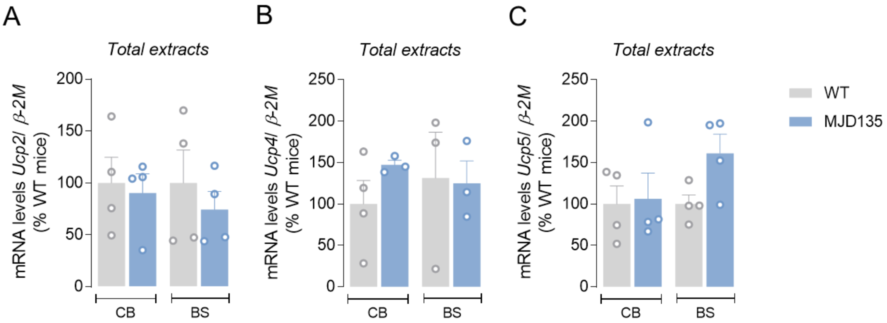

Figure S2. mRNA levels of *Ucp2/4/5* in mitochondrial or total extracts from cerebellum or brainstem. mRNA levels for *Ucp2* (**A**), *Ucp4* (**B**) and Ucp5 (**C**) were assessed in total extracts from CB and BS of MJD135 *versus* WT mice as described in methods section.

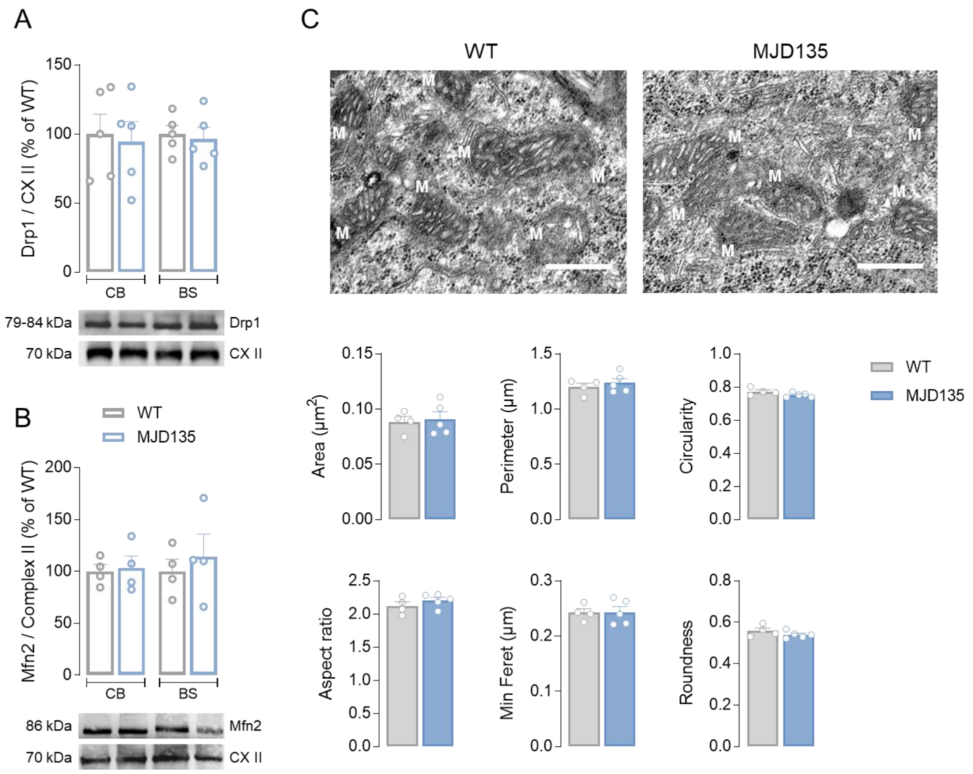

Figure S3. Mitochondrial Drp1 and Mfn2 protein levels and TEM analysis in cerebellum and brainstem of MJD135 *versus* WT mice. Protein levels of Drp1 (**A**) and Mfn2 (**B**) were determined in mitochondria isolated from the cerebellum and brainstem of MJD135 *versus* WT mice through Western Blotting. Cx II (70 kDa subunit) was used as internal loading control. Electron micrographs were obtained from MJD135 mice cerebellum and mitochondrial morphology presented for area, perimeter, circularity, aspect ratio, min Feret and roundness were evaluated by FIJI software. (**C**; scale bar: 0.5 µm). "M" denotes for mitochondria. Data are presented as mean ± SEM of 4-5 mice from each genotype.

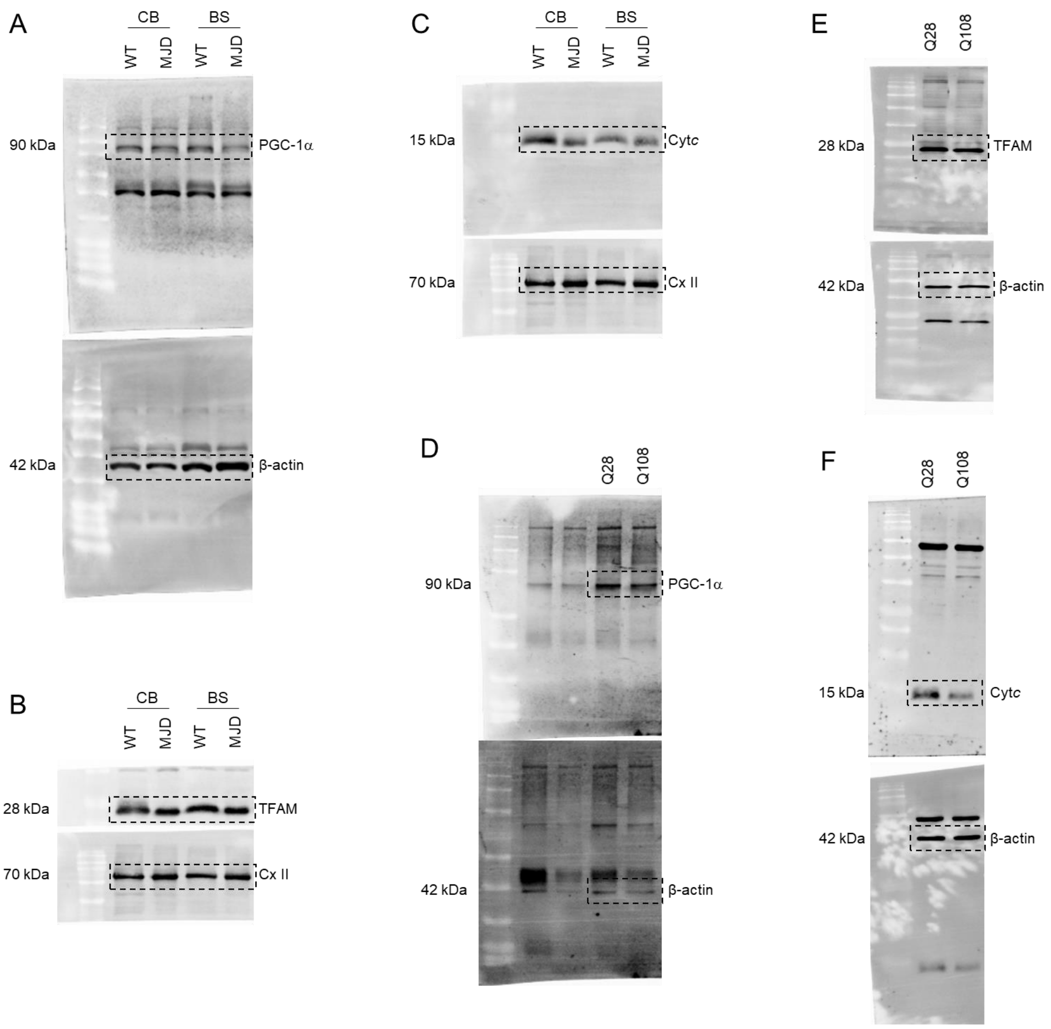

Figure S4. Full-length Western blots for PGC1-α, TFAM and cytochrome *c* in MJD135 mouse cerebellum and brainstem mitochondria and in PC6-3 cells. Full-length blot images for figure 4A (A), figure 4B (B), figure 4C (C), figure 4E (D), figure 4F (E) and figure 4G (F).

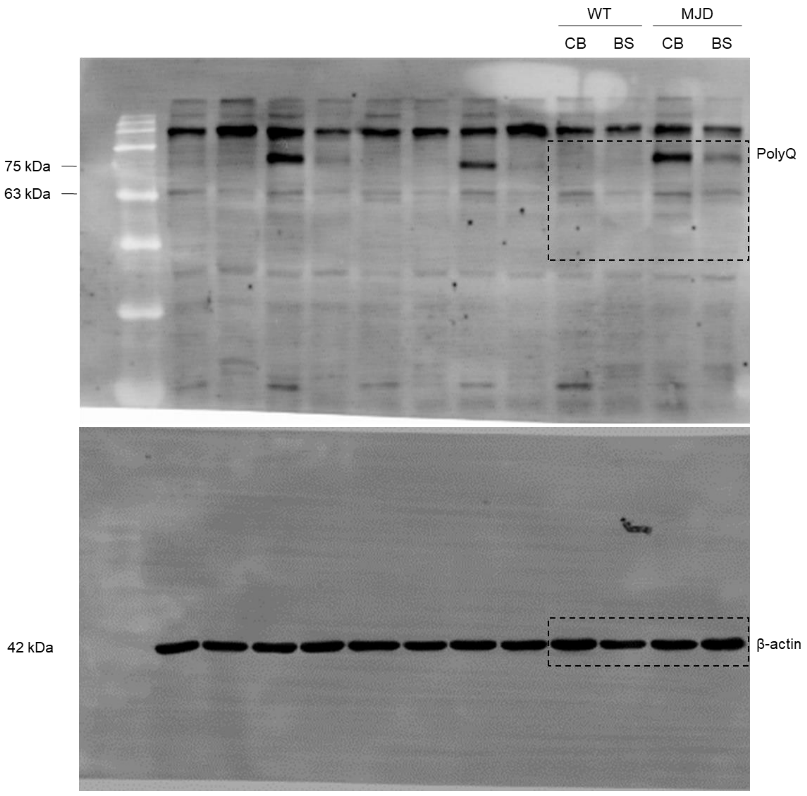

Figure S5. Full-length Western blots for mutant ATXN3 in MJD mice. Full-length blot images for figure S1C.

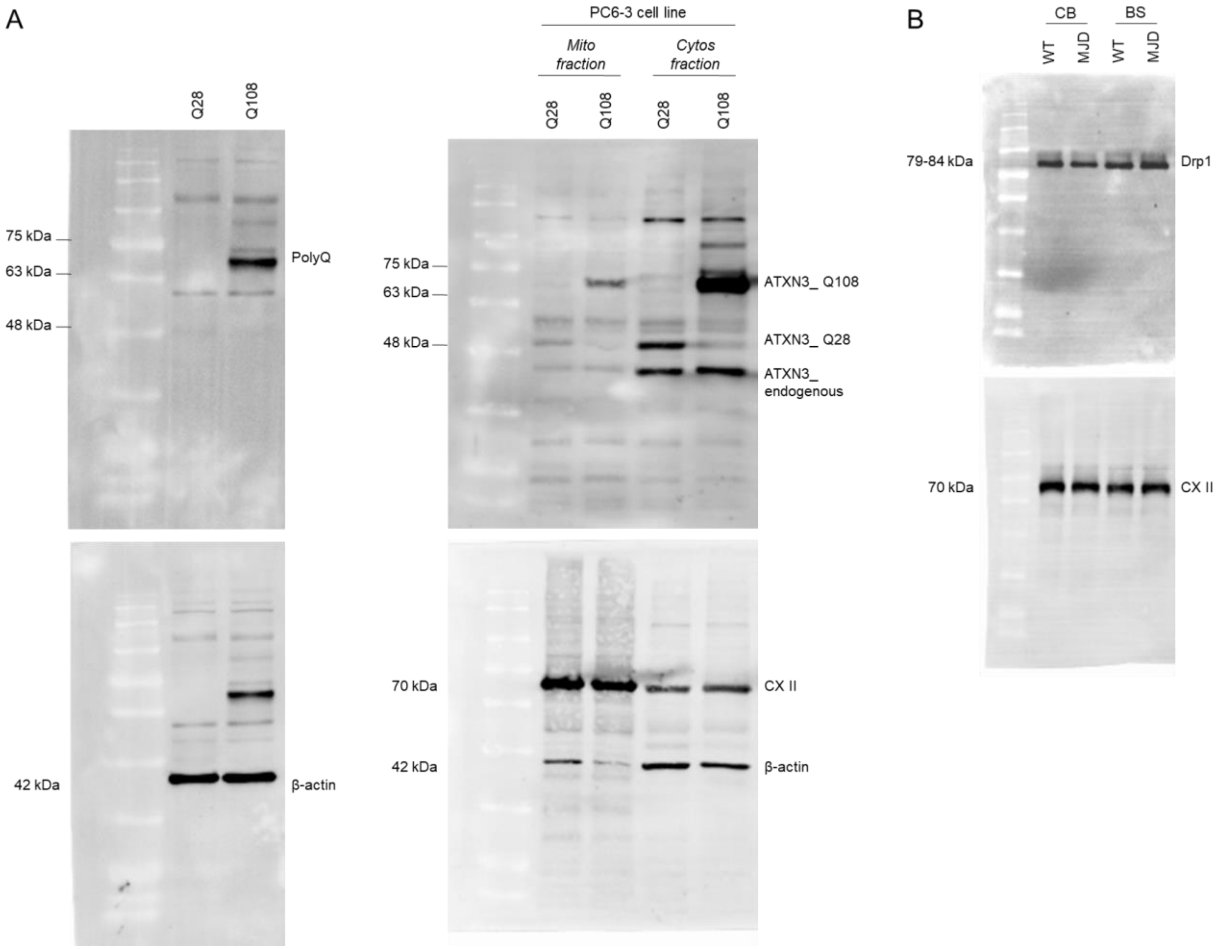

Figure S6. Full-length Western blots for mutant ATXN3 in PC6-3 cells and mitochondrial Drp1MJD135 mouse cerebellum and brainstem. Full-length blot images for figure S1D (A) and figure S3A (B).
